# O.6.3-4 Children’s Sport Participation and Physical Activity in the Island of Ireland: a cross-sectional study comparing differences in participation between 2018 and 2022

**DOI:** 10.1093/eurpub/ckad133.284

**Published:** 2023-09-11

**Authors:** Julia McClelland, Kwok Ng, Una Britton, Conor Philpott, Brendan O'Keeffe, Ali Sheikhi, Stephen Behan, Diarmuid Lester, Manolis Adamakis, Joao Costa, Tara Coppinger, Sinead Connolly, Hannah Goss, Wesley O'Brien, Paul McFlynn, Marie Murphy, SarahJane Belton, Catherine Woods

**Affiliations:** Ulster University, UK; University of Limerick, Ireland; Dublin City University, Ireland; University College Cork, Ireland; University of Limerick, Ireland; University of Limerick, Ireland; Dublin City University, Ireland; University College Cork, Ireland; University College Cork, Ireland; University College Cork, Ireland; Munster Technological University, Ireland; j.mcclelland1@ulster.ac.uk; Ulster University, UK; Dublin City University, Ireland; University College Cork, Ireland; Ulster University, UK; Ulster University, UK; Dublin City University, Ireland; University of Limerick, Ireland

## Abstract

**Purpose:**

Given the many policies and interventions aimed at increasing physical activity (PA), it is important to understand how levels of PA, sport participation and active travel change over time. The purpose of this study is to assess the participation levels of children and adolescents in sport, PA and active travel in Northern Ireland (NI) and the Republic of Ireland (RoI) and compare differences in participation between 2018 and 2022.

**Methods:**

A cross-sectional survey was completed by a sample of schoolchildren in 2018 and in 2022 through the *Children’s Sport Participation and Physical Activity (CSPPA) study*. A representative sample of primary (n = 1549 in 2018, n = 1758 in 2022) and post-primary (n = 5102 in 2018, n = 7123 in 2022) pupils were surveyed in school using standardised self-report PA and sport participation measures. Descriptive statistics, chi-square analyses, linear regression and logistic regression were run for outcome variables.

**Results:**

Reported participation in daily PA increased at both school levels in NI and RoI between 2018 and 2022 with an increased proportion of pupils meeting the recommended levels of PA (NI: 13% in 2018 and 17% in 2022, RoI: 13% in 2018 and 15% in 2022). The proportion of children taking part in sport at least once a week increased over the last four years at both school levels in NI and RoI. Active travel to or from primary school increased from 36% in 2018 to 40% in 2022 in NI and from 42% in 2018 to 43% in 2022 in RoI. There was no change in the level of self-reported active travel for post-primary pupils in NI. However, reported engagement in active travel decreased from 40% to 35% in RoI.

**Conclusions:**

The findings from this study suggest small increases in participation in self-reported daily PA, school sport, and community sport between 2018 and 2022. Despite this, participation rates and engagement with active travel remain low. These findings are important for policymakers seeking to address inactivity in children and adolescents in Ireland.

**Support/Funding Source:**

This study is funded by Sport Ireland, Healthy Ireland and Sport NI, with support from the Departments of Health and Education in ROI.

